# Developing the next generation of dissemination and implementation researchers: insights from initial trainees

**DOI:** 10.1186/1748-5908-8-29

**Published:** 2013-03-12

**Authors:** Katherine A Stamatakis, Wynne E Norton, Shannon W Stirman, Cathy Melvin, Ross C Brownson

**Affiliations:** 1 Division of Public Health Sciences and Alvin J. Siteman Cancer Center, Washington University School of Medicine, Washington University in St. Louis, St. Louis, MO, USA; 2 Department of Health Behavior, School of Public Health, University of Alabama at Birmingham, Birmingham, AL, USA; 3 Women's Health Sciences Division, National Center for PTSD, VA Boston Healthcare System, and Boston University, Boston, MA, USA; 4 Department of Medicine and Hollings Cancer Center, Medical University of South Carolina, Charleston, SC, USA; 5 Prevention Research Center in St. Louis, Brown School, Washington University in St. Louis, St. Louis, MO, USA

**Keywords:** Dissemination and implementation research, Career development, Early-stage investigators

## Abstract

**Background:**

Dissemination and implementation (D&I) research is a relatively young discipline, underscoring the importance of training and career development in building and sustaining the field. As such, D&I research faces several challenges in designing formal training programs and guidance for career development. A cohort of early-stage investigators (ESI) recently involved in an implementation research training program provided a resource for formative data in identifying needs and solutions around career development.

**Results:**

Responses outlined fellows’ perspectives on the perceived usefulness and importance of, as well as barriers to, developing practice linkages, acquiring additional methods training, academic advancement, and identifying institutional supports. Mentorship was a cross-cutting issue and was further discussed in terms of ways it could foster career advancement in the context of D&I research.

**Conclusions:**

Advancing an emerging field while simultaneously developing an academic career offers a unique challenge to ESIs in D&I research. This article summarizes findings from the formative data that outlines some directions for ESIs and provides linkages to the literature and other resources on key points.

## Background

Dissemination and implementation (D&I) research is a relatively young discipline, underscoring the importance of training and career development in building and sustaining the field [1]. As an emerging field built upon a transdisciplinary blend of sciences and oriented toward the application of research into practice settings, D&I research faces several challenges in designing formal training programs and guidance for career development: the evidence-base is small, though growing, with measures and methods still developing [2]; there are few established ‘experts’ and thus few senior mentors; institutional supports may be lacking; inconsistencies exist in terminology across settings and countries [3]; research often strays (by necessity and/or design) from the more widely-accepted randomized controlled trial [4,5]; and it often addresses highly intractable problems in complex systems that require team efforts [6]. Thus, the challenges for career development may extend beyond the need to enhance individual research competencies to a broader set of supports. However, these same challenges also point to opportunities for innovation in building a career in the field of D&I research. This brief report explores some issues in career development as described by a small cohort of early-stage investigators (ESI) in D&I research, serving as a source of formative data and providing linkages to the literature on key points.

### Perspectives from early-stage investigators

A cohort of ESI recently involved in an implementation research training program provided a resource for formative data around career development issues (http://cmhsr.wustl.edu/Training/IRI/Pages/ImplementationResearchTraining.aspx). In March of 2011, following attendance at the first summer training institute, we contacted participating fellows (n = 11), who were MD- and/or PhD-trained with a background in mental health research, and, via email, asked for responses to a brief series of five open-ended questions related to career development in D&I research (see Additional file 1). The questions were intended to catalyze discussion for a think-tank session on building the field of D&I research, presented at the 4^th^ Annual NIH Conference on the Science of Dissemination and Implementation. Responses (n = 8) to the series of five semi-structured, open-ended questions were generally brief (< 3 sentences) and were analyzed using principles of applied thematic analysis [7], with the analysis structured to outline fellows’ perspectives on the perceived usefulness, importance, barriers and solutions around career development issues, including: developing practice linkages, acquiring additional methods training, academic advancement, and identifying institutional supports (*e.g*., mentoring and technical assistance) (Table 1).

**Table 1:** Exemplary quotations from early stage investigators in dissemination and implementation research

**Domain**	**Quote**
Practice linkages	‘I think this is a key area, but it takes time to form these practice linkages, and they are not always seen as important in the academic world… I guess I'd say stick with it, as it can pay off in the long-term. I've always been focused on how my work has practical implications for service providers, so I think this focus has been immensely helpful in my work to date.’
Methods training	‘I attend various brief professional development seminars… [and] bring strong methods people into my projects to assist with these areas. It seems that the stats/methods areas are continually changing, and there is no way for me to maintain a state of the art focus on those areas, and my other content areas…so I work to keep my skills up, but collaborate with others who specialize in these areas.’
Technical assistance	‘This process of mentoring through NIH and IES [Institute of Educational Sciences] grant writing has been so valuable and has helped me become far more competitive....just the process of shaping ideas into reasonable and compelling grant proposals has been very helpful.’
Institutional supports	‘I think your home mentor is really the gatekeeper… [can offer] support for protected time to go off-site and meet with potential collaborators,… building a research program during the first three years, availability of research assistants, committee burden, supervision of doctoral students, travel/training budget.’
Academic advancement/ incentives	‘The name of the game is still publications and grant funding, so although practice collaborations may be recognized on some level, they will likely be most recognized if they are reflected in a publication or a strong grant application.’
Mentorship	‘It is important to connect and take advantage of mentors and colleagues who are fair, generous and resourceful. Be open to learning from other disciplines about theory and methodology when considering your implementation research. It is difficult to do D&I research by yourself, a team approach is key.’
Goal orientation	‘Although discouraging at times and lacking in immediate results remember you are building towards something that in the end is the toughest gap to bridge in science and can be very rewarding.’

### Developing practice linkages

Practice linkages are key to achieving the underlying D&I science goal of moving evidence to practice. Building upon well-established participatory approaches [8,9], it is essential that ESIs develop linkages to practice settings (*e.g*., clinics, health departments) and collaborations with community partners to facilitate their research. Early-stage investigators noted that guidance from faculty who have established such collaborations was critical in their development of strong practice linkages. Equally importantly, expert faculty can assist ESIs in their efforts to strike the right balance between activities that are essential to true collaborations, which are typically less valued by academic appointment and promotion committees [10], and the need to publish and obtain extramural funding. Senior investigators can pave the way for ESI development of strong practice linkages by making introductions, inviting ESIs to attend meetings with community partners, helping them to understand service system structures, policies and politics, and identifying mutually beneficial areas of collaboration.

### Methods training

Early stage D&I investigators identified additional training in research methods and designs as another key area for enhancing one’s D&I career. Often, given the challenges associated with D&I research, innovative and non-traditional research designs are employed [11]. Many fellows, however, noted that any single discipline rarely provides enough training and background in the variety of methods in D&I research studies, and highlighted the need for those seeking to develop a career in D&I science to gain additional training and experience in such approaches, including mixed-methods, qualitative methods, evaluation methods, comparative effectiveness research, pragmatic or practical clinical trials, quasi-experimental designs, and longitudinal analyses. Fellows suggested that researchers interested in developing and enhancing their skill-set in one or more of these methods seek out additional training opportunities through universities (*e.g*., professional development seminars), formal training programs (*e.g*., Canada Summer Institute on Knowledge Translation), conferences (*e.g*., pre-conference workshops), and cyber-seminar series (*e.g*., VA HSR&D cyber-seminar series). A selected list of online resources and training programs open to all those who apply across various health and practice settings are included in Table 2. Fellows also suggested seeking input from and collaborating with expert methodologists when designing D&I research studies.

**Table 2:** Select training programs, conferences and resources in dissemination and implementation science

**Type**	**Title**	**Brief description**	**Website/Information**
Training Programs	NIH Training Institute on Dissemination and Implementation Research in Health (TIDIRH)	Five-day training and mentoring focusing on all areas of health	http://conferences.thehillgroup.com/OBSSRinstitutes/TIDIRH2011/
	VA Quality Enhancement Research Initiative (QUERI) Enhancing Implementation Science (EIS) Conference	Recorded training conference plus follow-up cyber seminars addressing: overview of implementation science; theoretical frameworks and design of implementation programs; and evaluation designs and methods	http://www.queri.research.va.gov/ciprs/training.cfm
	Knowledge Translation Canada Summer Institute	Three-day program employing active learning strategies, mentorship opportunities, and peer-to-peer networking	http://ktclearinghouse.ca/ktcanada/education/summerinstitute
Conferences	NIH Conference on the Science of Dissemination and Implementation*	Two-day conference hosted by Department of Health and Human Services, National Institutes of Health, Office of Behavioral and Social Sciences Research, and the VA	http://obssr.od.nih.gov/scientific_areas/translation/dissemination_and_implementation/DI2012/index.html
	Global Implementation Conference	Biennial conference on the science and practice of using science in practice	http://www.implementationconference.org/
	Seattle Implementation Research Conference	Biennial conference on the implementation of psychosocial interventions, with an emphasis on tangible products and tools and developing junior colleagues	http://www.seattleimplementation.org
Resources	Dissemination and Implementation in Health e-Newsletter	Distributes late-breaking information on research, practice and policy activities in D&I in health care and public health	Contact wenorton@uab.edu for details
	VA QUERI Cyber Seminar Series	Cyber seminars addressing: overview of implementation science; theoretical frameworks and design of implementation programs; and evaluation designs and methods	http://www.hsrd.research.va.gov/cyberseminars/series.cfm
	National Implementation Research Network	Mission is to contribute to the best practices and science of implementation, organization change, and system reinvention to improve outcomes across the spectrum of human services	http://www.fpg.unc.edu/~nirn/
	Seattle Implementation Research Conference measures project	Development of a comprehensive library of D&I instruments measuring influences on implementation	http://www.seattleimplementation.org/sirc-projects/sirc-instrument-project/measures-collection/

* This conference was not held in 2013, though plans are underway for 2014 http://obssr.od.nih.gov/index.aspx.

### Academic advancement and incentives

As publications and grant funding are weighted heavily in academic performance reviews, ESIs need to be sure to find ways to translate the collaborations that they build with practice settings and the development of new methods into publications and strong grant applications. Because the development of strong practice linkages and the timeline for implementation studies can span several years, many commented on the importance of opportunities to publish using data that has previously been collected through an existing collaboration. Some stated that they had been able to find a niche within a larger ongoing implementation study that is untapped by other investigators. In the absence of such projects, other ESIs were able to identify areas in which a systematic review or pilot research could make a contribution to the field and lay the foundation for larger projects. ESIs indicated that they benefitted from guidance around the choice of appropriate and feasible funding, career development, and smaller grant funding mechanisms (*e.g*., Clinical and Translational Science Award pilot grants) to fund pilot research in order to build toward competing for larger, R01-type grant projects. Finally, some early-career investigators noted that they had found ways to leverage their expertise, which may be fairly rare at their university or within their department, to build collaborations with other investigators that will ultimately result in publications and funding.

### Institutional supports

Early-stage investigators emphasized the value of institutional support in advancing one’s D&I career. Institutional characteristics cited as being especially important to career development included having a strong history of funded mentoring programs (*e.g*., K-awards, T32 programs), senior researchers available and interested in mentoring junior faculty, travel funds, protected time — especially for developing working relationships with practice organizations and potential D&I research sites — and grant writing support. Moreover, having a mentor who is well connected to the D&I community and community partners — both within and outside of one’s own institution — is important, as she or he can provide linkages to potential collaborators and co-mentors to help gain additional experience in and exposure to cutting-edge research. While the need for mentoring and institutional supports is common to career development across many fields of research, features of the field of D&I research that may create a unique set of needs and opportunities are discussed below.

### Mentorship as a cross-cutting issue

The importance of mentorship and guidance from senior investigators was a recurring theme that cross-cut other issues discussed, likely reflecting the need for guidance to offset the uncertainty inherent in developing a research career in an emerging field. While there is a broad literature showing the importance of academic mentorship in the sciences in general [12], it may be particularly fundamental in building capacity in a new area of research such as D&I [1]. Mentorship of junior faculty in D&I research is a cross-cutting issue, certainly as far as the themes discussed above, since an effective mentor can help junior investigators initiate and strengthen practice linkages, guide and find sources for additional methods training, provide access to data, and advise on issues around academic advancement and potential funding sources. An experienced mentor may provide guidance toward applying useful theories, insights into D&I processes, and exemplary interventions from previous work [13]. Issues around mentoring in D&I research are likely to be mostly similar to those in other fields of scientific research, particularly those aimed at translational research [14,15]. One difference may be that there are not many mentors to foster the careers of the growing number of junior investigators in D&I research at the current early stage of the field’s growth. This potential imbalance may increase the burden on mentors such that the risks in time and effort end up outweighing the personal and professional benefits [16]. However, the nature of research in D&I, which requires links with practice settings and across disciplines, also calls for the need to find mentorship from more than one source to guide specific areas of the research. For example, in addition to a D&I investigator, mentorship from an experienced practitioner-advisor can uniquely prepare ESIs for D&I research.

Another important dimension to mentorship involves advancing the careers of members of underrepresented groups in the scientific community, especially given the prominence of reaching underserved and disparately burdened populations as a priority for D&I research [17]. There is some evidence that for minorities, having a mentor of the same demographic background is beneficial [18], although successful mentorship can be achieved across racial and other divides and may benefit from training in providing guidance and thoughtful critical feedback [19].

### Context for future directions

The focus of this article is primarily on research situated in US academic settings, though many of the ideas may be applicable to other research settings, geographic areas, and funding sources [20]. Given the background of the current study sample, these findings may be most relevant to ESIs in academic medical centers with traditional promotion policies. We found some common themes with previous work that identified challenges for implementation scientists working in a large, healthcare system [21]. Future work building upon the current study could aim to include a broader sample of ESIs (*e.g*., not limited to mental health research) as well as senior investigators, who may offer a different perspective on training needs and institutional supports for career advancement. To provide some context for the current cohort of D&I researchers in the US, we examined the rank and gender of the 50 principal investigators (PI) funded through the NIH Program Announcement for Dissemination and Implementation Research in Health, which included R01, R21 and R03 mechanisms [22]. Of the 41 funded PIs whose academic ranking was ascertainable from their academic website, most were mid-to-senior ranked, with only 19.5% assistant professors (Table 3).

**Table 3:** Characteristics (academic ranking and gender) of NIH-funded principal investigators* in dissemination and implementation research

**Rank**	**Male**	**Female**	**Total**
	**% (freq)**	**% (freq)**	**% (freq)**
Full	61.1 (11)	38.9 (7)	43.9 (18)
Associate	40.0 (6)	60.0 (9)	36.6 (15)
Assistant	25.0 (2)	75.0 (6)	19.5 (8)
Total	46.3 (19)	53.7 (22)	41

*As listed at http://obssr.od.nih.gov/scientific_areas/translation/dissemination_and_implementation/index.aspx as of 11/15/11.

Advancing an emerging field while simultaneously developing an academic career offers a unique challenge to ESIs in D&I research. We hope this paper outlines some directions for future investigations into building a stronger career foundation that may include developing practice linkages, filling additional methods training, identifying institutional supports (particularly around academic advancement), and strategically seeking training and guidance from senior mentors. As we have learned from related fields, the adage ‘all teach, all learn, all improve’ is an important guiding philosophy [23], and strengthening linkages among D&I researchers at all career stages is an implicit — and worthy — goal.

## Abbreviations

D&I: Dissemination and implementation; NIH: National Institutes of Health; KT: Knowledge translation; VA: Veterans Administration; NIMH: National Institute of Mental Health; IRI: Implementation Research Institute; TIDIRH: Training Institute on Dissemination and Implementation Research in Health

## Competing interests

Wynne E. Norton is on the Editorial Board of *Implementation Science*. There are no other competing interests to report.

## Authors’ contributions

KAS contributed to the study design, carried out the content analysis, supervised all other data analysis, and drafted the manuscript. WEN and SWS contributed to the analysis, writing the draft and reviewing the manuscript. CM contributed to writing and reviewing the manuscript. RCB coordinated the study development and contributed to writing and reviewing the manuscript. All authors read and approved the final manuscript.

## Supplementary Material

Additional file 1 Questions for semi-structured interview. Click here for file
