# Supplementary material for: Developing the next generation of dissemination and implementation researchers: insights from initial trainees
Source: Implement Sci. 2013 Mar 12;8:29. doi: 10.1186/1748-5908-8-29 (PMC3626831; doi:10.1186/1748-5908-8-29)
Supplement: Additional file 1 — Questions for semi-structured interview. [file 1748-5908-8-29-S1.docx]

Additional File 1: Questions for semi-structured interview

1) What does a junior faculty member need to do as an individual investigator to develop strong practice linkages?

2) What additional methods training have you sought?

3) What institutional supports should one look for/ask for in terms of mentoring and technical assistance?

4) Do you have advice for other junior investigators related to academic advancement and/or tenure (e.g., will the effort spent developing practice collaborations be recognized?)?

5) Based on your experiences thus far in your career, what other advice would you give to junior D&I researchers who seek to build an academic career?
